# An Integrated Approach for Identifying Molecular Subtypes in Human Colon Cancer Using Gene Expression Data

**DOI:** 10.3390/genes9080397

**Published:** 2018-08-02

**Authors:** Wen-Hui Wang, Ting-Yan Xie, Guang-Lei Xie, Zhong-Lu Ren, Jin-Ming Li

**Affiliations:** 1 State Key Laboratory of Organ Failure Research, Division of Nephrology, Southern Medical University, Guangzhou 510515, China; thineeyes@163.com (W.-H.W.); bobanne@163.com (T.-Y.X.); xgl343@smu.edu.cn (G.-L.X.); 2 Department of Bioinformatics, School of Basic Medical Sciences, Southern Medical University, Guangzhou 510515, China; 3 Network Information Center, The Sixth Affiliated Hospital of Sun Yat-Sen University, Guangzhou 510655, China; 4 Center for Systems Medical Genetics, Department of Obstetrics & Gynecology Nanfang Hospital, Southern Medical University, Guangzhou 510515, China; renzhonglu@smu.edu.cn; 5 Laboratory of Systems Neuroscience, Institute of Mental Health Southern Medical University, Southern Medical University, Guangzhou 510515, China

**Keywords:** subtypes of cancer, colon cancer, Bayesian robust principal component, hierarchical clustering, feature selection

## Abstract

Identifying molecular subtypes of colorectal cancer (CRC) may allow for more rational, patient-specific treatment. Various studies have identified molecular subtypes for CRC using gene expression data, but they are inconsistent and further research is necessary. From a methodological point of view, a progressive approach is needed to identify molecular subtypes in human colon cancer using gene expression data. We propose an approach to identify the molecular subtypes of colon cancer that integrates denoising by the Bayesian robust principal component analysis (BRPCA) algorithm, hierarchical clustering by the directed bubble hierarchical tree (DBHT) algorithm, and feature gene selection by an improved differential evolution based feature selection method (DEFS_W_) algorithm. In this approach, the normal samples being completely and exclusively clustered into one class is considered to be the standard of reasonable clustering subtypes, and the feature selection pays attention to imbalances of samples among subtypes. With this approach, we identified the molecular subtypes of colon cancer on the mRNA gene expression dataset of 153 colon cancer samples and 19 normal control samples of the Cancer Genome Atlas (TCGA) project. The colon cancer was clustered into 7 subtypes with 44 feature genes. Our approach could identify finer subtypes of colon cancer with fewer feature genes than the other two recent studies and exhibits a generic methodology that might be applied to identify the subtypes of other cancers.

## 1. Introduction

Identifying the molecular subtypes of colorectal cancer (CRC) may allow for more rational, patient-specific treatment in the future. Various studies have been done to predict molecular subtypes for CRC based on gene expression data. Fearon and Vogelstein utilized four different genomic and epigenomic instabilities to identify four subtypes of CRC: chromosome instability (CIN), microsatellite instability (MSI), CpG island methylator phenotype (CIMP), and DNA global hypomethylation [1]. Using consensus clustering based on self-organizing maps, nearest centroid classifier, and hierarchical clustering, Muzny et al. showed that CRC has MSI/CIMP, CIN, and invasive subtypes with 1020 signature genes (340 genes per class) at the gene expression level [2]. The Colorectal Cancer Subtyping Consortium found our consensus molecular subtypes (CMSs) among six independent classification systems [3]. However, there remained 13% “mixed or indeterminate” samples that had heterogeneous patterns of CMS mixtures but did not constitute a fifth subtype [4]. Ren et al. utilized consensus clustering based on K-means to identify the ECL1 and ECL2 subtypes of colon cancer and further classify the ECL1 into three subclasses [5]. These subtypes of CRC found in previous studies appear to be inconsistent, and further research is necessary. From a methodological point of view, a progressive approach is needed to identify the finer subtypes.

One popular approach to identifying cancer subtypes is clustering the gene expression data of patient samples, as expression data can give a comprehensive snapshot of transcription activities for whole genomes [6]. Because of the intrinsic noise of the gene expression data generated using microarray or high-throughput sequencing technology, it is desirable to remove noise before clustering. The usual method is to project gene expressions onto a small number of principal components (PCs) by principal component analysis (PCA), since the first few principal components can usually capture most of the variations in gene expressions, whereas the rest of the PCs are often assumed to capture only the residual noise. However, choosing the proper number of PCs remains an open problem [7]. Recently, a new method for matrix recovery, Bayesian robust PCA (BRPCA), was introduced in the field of image processing [8]. It decomposes an observed matrix into low-rank, sparse, and noise components. The gene expression data all lie near some low-dimensional subspaces, so it is natural to treat those genes of nondifferential expression as approximately low rank and those with differential expression as sparse perturbation signals [9]. With the noise and smooth low-rank signals filtered by expression profiling, the sparse components are undoubtedly the perfect signals for identifying the subtypes by clustering similar samples. The usual clustering method is unsupervised clustering, which avoids defining the number of subtypes. Song et al. proposed hierarchical information clustering by means of topologically embedded graphs (named DBHT for short), which does not need any parameters and outperformed some of the state-of-the-art cluster analysis techniques with the best parameter settings, such as Kmeans++, spectral clustering via normalized cut on k-nearest neighbor graph (kNN-Spectral), self-organizing map (SOM), and Q-cut [10]. DBHT is a graph-theoretic approach to extracting clusters and hierarchies in complex datasets in an unsupervised and deterministic manner, without the use of any prior information. For gene expression data, this method provides both the intracluster hierarchy, which describes the way clusters are composed, and the intercluster hierarchy, which describes how clusters gather together. On one side, clustering the samples into subtypes is done on the premise that the samples are cancer samples; on the other side, the BRPCA needs to tune its hyperparameter settings. Therefore, we draw in the concept of “reference object” from classical physics. Before doing the BRPCA analysis, we add some normal samples as the reference objects. Only when the normal samples are correctly clustered together do we consider the clustering reasonable in identifying the subtypes. After identifying the subtypes, getting the marker genes is a very important task. The DBHT algorithm can also be used to extract significantly differentiating gene groups among subtypes to select the feature genes for sample classification. However, it is not suitable for large-scale genomic data due to several drawbacks [11]. In our previous study, we used consensus clustering to identify subtypes of colon cancer and got 256 feature genes [4]. Hundreds of feature genes distinctly hamper the translation to clinical practice. Therefore, more efficient and effective methods should be developed to select the feature genes that discriminate the subgroups at the top level. Recently, several new algorithms for feature selection have been proposed [12,13,14,15,16,17,18,19,20]. Our study suggests that the methods based on differential evolution (DE) in [17,18] can achieve remarkably good results compared with other well-known feature selection methods. Al-Ani et al. proposed the differential evolution based feature selection method (DEFS_W_) method [18], which not only is able to select feature subsets with a predefined cardinality (which is its main functionality), but also can discover the optimal feature subset size. A wrapper classifier is needed in the DEFS_W_ algorithm. In view of the usual imbalance of samples among subtypes, we use the naive Bayes (NB) classifier with empirical prior probabilities and weight accuracy to evaluate classification ability. The empirical prior probabilities estimate the prior probabilities from the relative frequencies of the classes in training, which can lessen the influence of the imbalance of samples. Weight accuracy is a special assessment measurement for classification of imbalance samples.

In this study, we integrated these state-of-the-art techniques of denoising, clustering, and feature selection to identify molecular subtypes in human colon cancer using gene expression data. Our integrated approach incorporates denoising by the BRPCA, hierarchical clustering by the DBHT, and selecting feature genes by DEFS_W_. We applied this approach to the Cancer Genome Atlas (TCGA, http://cancergenome.nih.gov/) mRNA gene expression dataset of colon cancer and identified 7 subtypes with 44 feature genes. The results deliver finer subtyping with fewer feature genes than in the other two recent studies.

## 2. Materials and Methods

### 2.1. Dataset

The microarray mRNA gene expression dataset we used to identify the subtypes of colon cancer is from TCGA. It includes 153 cancers samples, which have been used by Muzny et al. [2] and Ren et al. [4] for the same purpose, and 19 control normal samples. We used the level 3 dataset from TCGA, and this was downloaded by the R package “RTCGA” [21].

### 2.2. Method Overview

**Figure 1:**
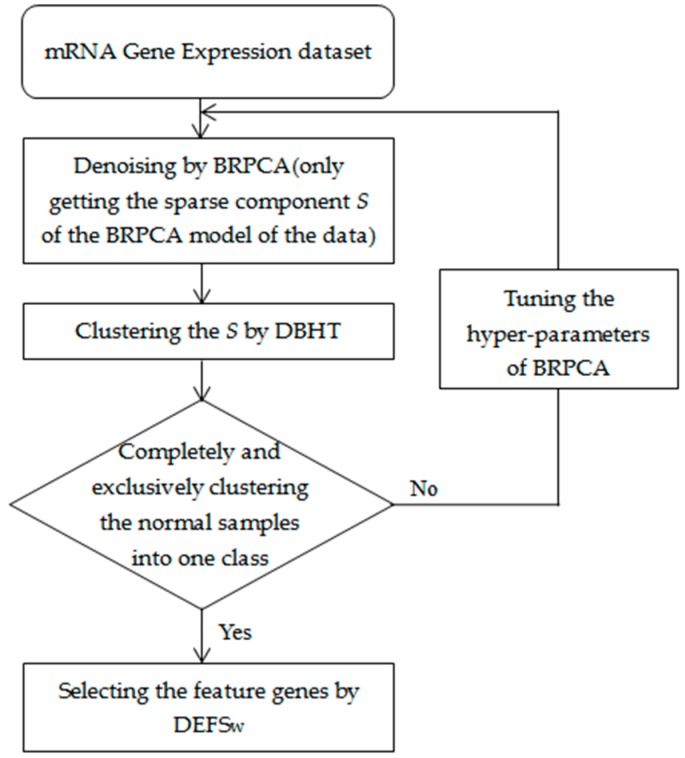
Summary of our integrative approach. BRPCA: Bayesian robust principal component analysis; DBHT: directed bubble hierarchical tree; DEFSw: differential evolution based feature selection.

We first applied the BRPCA to denoise the gene expression data by getting the sparse component but removing the low-rank and noise components, and then we used the DBHT to cluster the sparse components in the BRPCA. The normal samples completely and exclusively clustered into one class were considered as the standard of reasonable clustering. If the standard was not reached, we continually tuned the setting of the hyperparameters of the BRPCA until the clustering was up to the standard. Finally, we used the DEFS_W_ to select the feature genes for the clusters. A summary of our approach is shown in Figure 1. The BRPCA, DBHT and DEFSw algorithms were developed in MATLAB (MathWorks, Natick, MA, USA), and all the related source codes for implementing our approach are available in the Supplementary file 2 with a brief README.

#### 2.2.1. Bayesian Robust Principal Component Analysis

In the BRPCA model [8], the observed data matrix $Y\in {\mathbb{R}}^{P\times N}$ is the superposition of 3 parts: low-rank component $L\in R^{P\times N}$, sparse component $S\in {\mathbb{R}}^{P\times N}$, and noise term $E\in {\mathbb{R}}^{P\times N}$,

(1) Y=L+S+E

where P is the number of genes, and N is the number of samples. Furthermore,

(2) L=D(ZΛ)W, S=B∘X

where $\Lambda \in {\mathbb{R}}^{K\times K}$ is a diagonal matrix, $X\in {\mathbb{R}}^{P\times N}$, and ∘ denotes the pointwise product. The diagonal matrix Z has binary entries along the diagonal, $z_{kk}\in \{0,1\}$ for k=1,…K and the binary matrix $B\in {\left\{0,1\right\}}^{P\times N}$ is sparse. The integer K defines the largest possible rank that may be inferred for L. The BRPCA model assumed:

(3) $$d_{k}∼N\left(0,\frac{1}{P}I_{P}\right),\;k=1,\ldots K,\;D=[d_{1},\ldots d_{K}]$$

(4) $$w_{n}∼N\left(0,\frac{1}{K}I_{K}\right),\;n=1,\ldots N,\;W=[w_{1},\ldots w_{N}]$$

(5) $$\lambda_{kk}∼N(0,\tau^{-1}),k=1,\cdots K,\;\tau ∼\mathrm{Gamma}(a_{0},b_{0}),\;\Lambda =\mathrm{diag}[\lambda_{1},\ldots \lambda_{k}]$$

(6) $$z_{kk}∼\mathrm{Bernoulli}(p_{k}),\;p_{k}∼\mathrm{Beta}(\alpha_{0},\beta_{0}),k=1,\cdots K$$

(7) $$b_{i}∼\prod_{p=1}^{P}\mathrm{Bernoulli}(\pi_{pi}),\;i=1,\ldots N,B=[b_{1},\ldots b_{N}]$$

(8) $$x_{N}∼N(0,\nu^{-1}I_{N}),n=1,\ldots N,\nu ∼\mathrm{Gamma}(c_{0},d_{0}),\;X=[x_{1},\ldots x_{N}]$$

(9) $$e_{pn}∼N(0,\gamma_{n}^{-1}),\;p=1,\ldots P,\;\gamma_{n}∼\mathrm{Gamma}(e_{0},f_{0})\;$$

In our study, the observed data matrix $Y\in {\mathbb{R}}^{P\times N}$ was gene expression profiling of colon cancer with P=17,814 genes and N=172 samples (153 cancer samples and 19 normal samples). Each column is a gene expression profile and each row is the gene expression data in every sample. Different from image processing, which the BRPCA was originally used for [8], our data matrix Y consists of 2 types of columns, tumor samples and normal samples, and most genes across 2 types of samples should share some common characteristics. Therefore, we assume the appearance of the sparse component across sample (column) satisfies a Markov property, i.e.,

(10) $$\pi_{pi}\;∼\;\{\begin{array}{l} \mathrm{Beta}(\alpha_{H},\beta_{H})\;\mathrm{if}\;[0.5b_{p,i}+0.25(b_{p,i-1}+b_{p,i+1})]\geq 0.5\\ \mathrm{Beta}(\alpha_{L},\beta_{L})\;\mathrm{if}\;[0.5b_{p,i}+0.25(b_{p,i-1}+b_{p,i+1})]<0.5 \end{array}\;$$

where p=1,…P,i=2,…N−1.

For i=1, N, we may use sample 2 and sample N−1 twice, respectively, since sample 1 has no left neighbor and sample N has no right neighbor. Specifically, a gene with high expression in the left and right neighbors of a sample should have a high probability of expressing highly in this sample; hence the sparsity of a sample depends on its neighbors.

Since the density function at one layer is conjugate to the density function at the layer above it, the posterior density function is easily computed via Markov chain Monte Carlo (MCMC) implemented using a Gibbs sampler. The details for calculation of the BRPCA algorithm are described in algorithm 1 in Ding et al. [8].

#### 2.2.2. Hierarchical Information Clustering by Means of Topologically Embedded Graphs

The directed bubble hierarchical tree (DBHT) [10] algorithm is used to extract cluster structure and detect hierarchical organization in complex datasets. This approach is based on the properties of topologically embedded graphs built from a similarity measure. The general idea of the DBHT is to use the topological structure of a planar maximally filtered graph (PMFG) [22] to investigate the properties of the datasets. PMFG is a triangulation of a topological sphere. It has been shown that PMFG graphs are efficient filtering tools, with topological properties associated with the properties of the underlying system [22,23]. This makes the PMFG a desirable tool to extract clusters and hierarchies from complex datasets.

In our study, a sample is a vertex and the Pearson’s correlation coefficient matrix is used as the similarity matrix of the vertexes.

(11) $$r=\frac{\sum_{i=1}^{p}\left(x_{i}-\bar{x})(y_{i}-\bar{y}\right)}{\sqrt{\sum_{i=1}^{p}{\left(y_{i}-\bar{x}\right)}^{2}{\left(y_{i}-\bar{y}\right)}^{2}}}\;$$

The dissimilarity matrix of the vertexes we used is:

(12) $$d=\sqrt{2\times (1-r)}$$

Based on the similarity and dissimilarity matrix of samples, the DBHT constructs the PMFG to perform clustering and get hierarchies of samples. The details for the DBHT algorithm are described in Song et al. [10].

#### 2.2.3. Differential Evolution Based Feature Selection Method

To identify the subtypes using gene expression profiling, feature selection can be used to reduce the high-dimensionality of huge amounts of otherwise meaningless data. Khushaba and Al-Ani et al. proposed a powerful feature selection method that utilizes the differential evolution (DE) float number optimizer in the combinatorial optimization problem of feature selection, named DEFS_O_ [17], followed by an improved version, DEFS_W_ [18]. In the DEFS_O_, the desired feature subset size is predefined by the user, while in the DEFS_W_, the optimal feature subset size can be discovered automatically only by setting an upper limit. In the two algorithms, a wrapper classifier such as K nearest neighbor (KNN), support vector machine (SVM), or naive Bayes (NB) classifier is needed. The wrapper classifier and an assessment measurement such as classification accuracy are used together to evaluate the classification ability of features.

**Figure 2:**
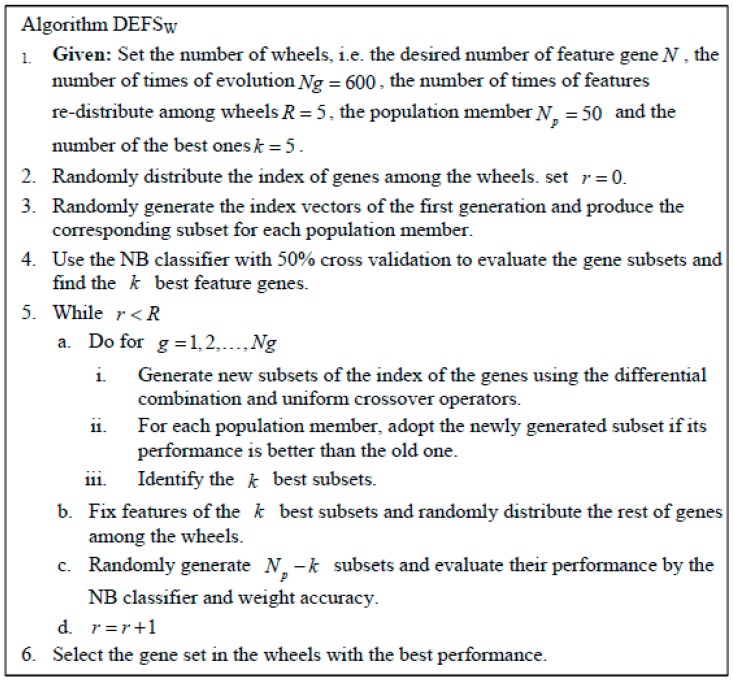
The DEFS_W_ algorithm and its parameter setting in our study.

In our study, some subtypes have a small number of samples and others have a lot, i.e., there are imbalances of samples among subtypes. Therefore, a wrapper classifier and an assessment measurement that can cope with the class imbalance have to be used to avoid the “larger subtypes win.” Meanwhile, for the DE algorithm, the computation cost is generally huge because of its iterative evolution, so a fast and simple classifier is desired. Not only can the NB classifier be trained very efficiently under the condition of a small amount of training data and take only linear time, but its empirical prior probabilities can lessen the influence of the imbalance of samples. To assess classification ability, we used weight accuracy instead of the usual classification accuracy. The weight accuracy of classification is defined by Draminski et al. [20] as:

(13) $$wAcc=\frac{1}{c}\sum_{i=1}^{c}\frac{n_{ii}}{n_{i1}+n_{i2}+\cdots n_{ic}}$$

where c is the number of classes, and $n_{ij}$ denotes the number of samples in the class i classified as those from class j. The wAcc considers sizes of classes in such a way as to prevent undue influence of a majority class on the performance index, and can more effectively assess the ability to classify the selected feature genes in the imbalanced data. The DEFS_W_ algorithm and its parameter setting that we used are listed in Figure 2.

## 3. Results

**Figure 3:**
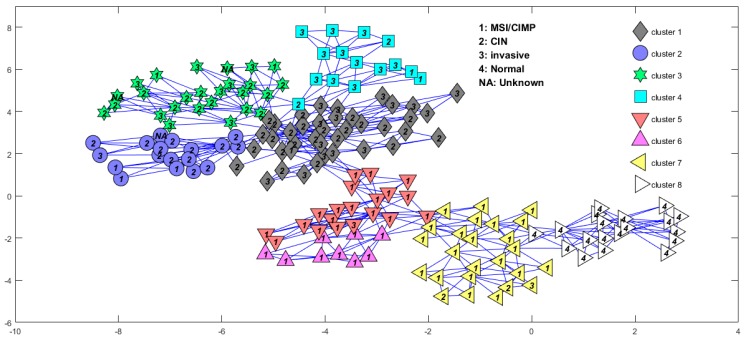
Sample cluster structure from directed bubble hierarchical tree (DBHT) analysis of the sparse component in the BRPCA (Bayesian robust principal component analysis) model for the 153 colon cancer samples and 19 normal samples downloaded from the Cancer Genome Atlas (TCGA). The labels inside the symbols correspond to the different subtypes identified by Muzny et al. [2]. MSI: microsatellite instability; CIMP: CpG island methylator phenotype; CIN: chromosomal instability.

We first used the BRPCA to denoise. In the BRPCA model, we set the largest possible rank as k=30 and the model hyperparameters were finally specified as follows: $a_{0}=b_{0}=c_{0}=d_{0}=e_{0}=f_{0}=10^{-6}$, $\alpha_{0}=1/K$, $\beta_{0}=(K-1)/K$, and $\alpha_{H}=0.01P,\;\beta_{H}=0.99P$ $\alpha_{L}=0.99P,\;\beta_{L}=0.01P$. The initial values of the main arguments were set as $\nu =10^{-6},\;\gamma_{n}=1$. For MCMC-based Bayesian inference, the number of burn-in iterations $N_{burn-in}$ and collection iterations $N_{collect}$ were set as 200 and 100, respectively. Then we applied the DBHT to the sparse component S and obtained eight sample clusters (see Figure 3), in which the normal samples were completely and exclusively clustered into one cluster and most of the MSI/CIMP samples were divided into three subtypes and some parts of the “invasive” samples were clustered together. The confusion matrix [24] of the two kinds of subtypes is shown in Table 1. Each row of the matrix represents the samples in a predicted class by the method of Muzny et al. [2], while each column represents the samples in a predicted class by our method. We also compared the subtypes predicted by our method and Ren’s method. The confusion matrix of the two methods is shown in Table 2. It shows that the subtypes ECL1 and ECL2 identified by Ren can be further subdivided by our method.

**Figure 4:**
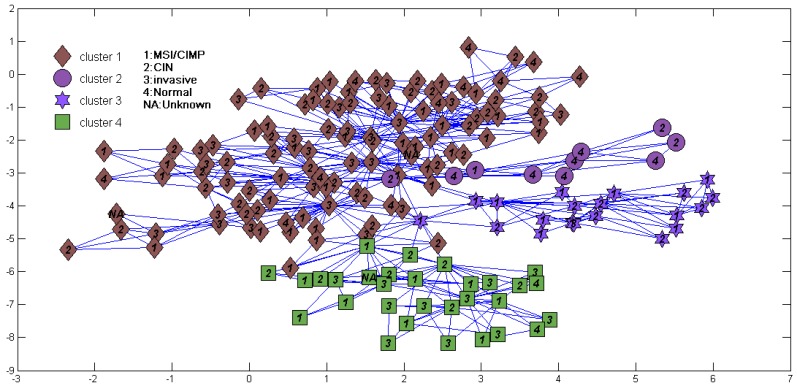
Sample cluster structure directly clustered using directed bubble hierarchical tree (DBHT) for the same data in Figure 3. Labels and symbols are also the same as Figure 3.

**Figure 5:**
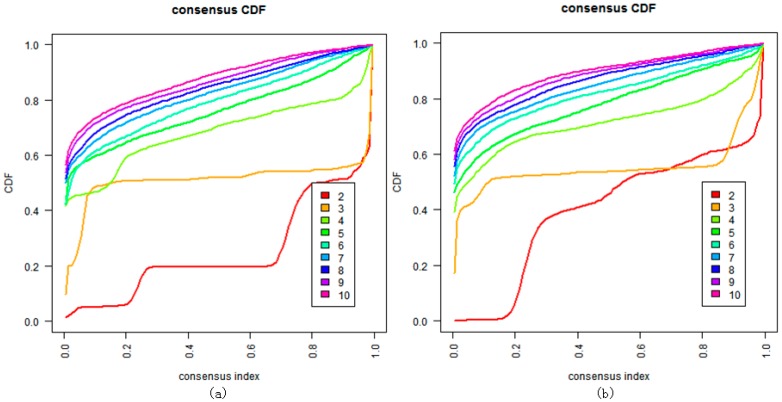
Clustering by the consensus clustering algorithm when *K* = 2 to 10. (**a**) Cluster consensus values and consensus cumulative distribution function (CDF) on the mRNA genes expression dataset; (**b**) cluster consensus values and consensus CDF on component S of the mRNA gene expression dataset.

We also tried to directly cluster the mRNA gene expression dataset using DBHT without denoising (Figure 4). It suggested that directly clustering by DBHT could not get any meaningful result; even the normal samples could not be clustered into one single class. We also did consensus hierarchical clustering [25] in the same way as that described in Ren et al. [4], and the result (Figure 5a) suggested that the samples were clustered into two clusters (normal and cancer samples) or three clusters (normal cluster, and ECL1 and ECL2 subtypes identified by Ren et al.). Using the same consensus clustering method, we also clustered component S of the samples in the BRPCA model, and the result (Figure 5b) suggested that consensus clustering could not get finer clusters for component S than the DBHT algorithm. All these suggested that the combination of BRPCA and DBHT could not only correctly cluster the normal samples, but also cluster the cancer samples into finer subtypes.

We then selected the feature genes for the identified subtypes by the DEFS_w_ algorithm. Considering that the latent premise of identifying subtypes is that the samples are from cancer patients, we expected that the selected feature genes could not only identify cancer subtypes, but also discriminate between tumor and normal samples, i.e., we expected to select out the feature genes that could discriminate all eight sample clusters. It was rational to select the feature genes from the differentially expressed genes (DEGs) of tumor vs. normal samples. We obtained 5897 DEGs by *t*-test (BH-correction, *p* value < 0.05) and fold change cutoff 1.5, and then selected the feature genes from these DEGs using the DEFS_W_ algorithm described in Figure 2. We set the upper limit of the size of feature genes from 100 to 20, and for each upper limit, the DEFS_w_ algorithm could deliver an optimal size of feature genes. We then determined our final size of feature genes to be that which gave the maximal average weight accuracy of the eight sample clusters. In this way, we ended up with 44 feature genes: *THBS2*, *NOX4*, *KIAA1199*, *SLC16A4*, *CCDC19*, *ZNRF3, GOLT1A*, *HYAL3*, *C15orf26*, *KIFC1*, *TIPIN*, *CTNNAL1*, *CALU*, *TAF1A*, *MCM2*, *MSH6*, *FLAD1*, *GCG*, *SCRG1*, *PTGER2*, *TIMD4*, *MUC1*, *PLOD2*, *LIMS2*, *ADH1B*, *PTN*, *PTPN7*, *AQP1*, *PSD3*, *CRAT*, *ATOH8*, *CGN*, *C6orf204*, *FTHP1*, *KCNMB1*, *LIG4*, *PPFIBP2*, *PPP2CB*, *ALAS2*, *ZZEF1, ATXN7*, *GRLF1*, *FAM102A*, and *C1orf152*. Among them, *MSH6* is known to be related to CRC, and is located in the Kyoto Encyclopedia of Genes and Genomes (KEGG) pathway of CRC [26]; *THBS2* is a potential prognostic biomarker in CRC [27] and can be used as an early diagnosis biomarkers of CRC [28]; Overexpression of *NOX4* predicts poor prognosis and promotes tumor progression in human CRC [29], and *NOX4* plays a role in PhIP-induced colon carcinogenesis, especially during the early stages before tumor onset [30], and moreover *NOX4* is highly predictive of relapse in stage II left-side colon cancer [31]; *KIAA1199* in human colorectal tumors (benign and malignant) is markedly higher than that in the normal colonic mucosa [32,33] and its overexpression promotes CRC cell migration and invasion [34], and could be used as a prognostic factor and novel therapeutic target for CRC [35]; Furthermore *KIAA1199* plays a critical role in maintaining an aggressive phenotype of tumor cells, and suppression of *KIAA1199*-related motilities of tumor cells contributes to reduced tumor metastasis in CRC [36]; *MCM2* is correlated with the cell proliferation state in colon cancer [37] and is more sensitive than *Ki-67* in identifying colorectal mucosal proliferation [38]; *MUC1* is aberrantly overexpressed in human colon cancers and is associated with invasion, metastases and a poor prognosis [39,40]; *ZNRF3* is important in serrated tumorigenesis and has identified a potential therapeutic strategy for CRC subtype [41]; *LIG4* may represent a new epigenetic marker for CRC independent of known markers [42]; *ADH1B* displays decreased expression during progression from adenoma to early and more advanced stage of colorectal carcinomas [43]. In contrast, Muzny et al. [2] did not select the feature genes for the identified subtypes, and Ren et al. [4] detected 256 genes as the marker genes of the ECL1 and ECL2 subtypes by Prediction Analysis of Microarrays (PAM) [44].

We used the same NB classifier to validate the classification ability of these feature genes. We did 10%, 20%, and 30% cross-validation (CV) with 1000 repeats. The mean classification accuracy, the mean weight accuracy of classification, and the mean classification accuracy for each class of the 1000 CVs are listed in Table 3. S1–S8 denotes the clusters 1–8 and S8 is the cluster of normal samples. The classification accuracy Acc depends on the number of samples correctly classified and is evaluated by the formula:

(14) $$Acc=\frac{t}{n}$$

where t is the number of samples correctly classified and n is the total number of samples.

## 4. Discussion

To identify cancer subtypes based on gene expression data, the proposed approach innovatively integrated state-of-art denoising, clustering, and feature selection algorithms. In BRPCA, the low-rank component of gene expression data may be the same as the background of an image and the noise component simulates unknown and nonstationary noise, whereas the sparse component may be the same as the foreground and is the key information for clustering. The DBHT is intrinsically a correlation-based clustering method. Through building the PMFG, the method does not need any prior tuning and provides both intracluster hierarchy, which describes the way clusters are composed, and intercluster hierarchy, which describes how clusters gather together. To assess whether the clustering is meaningful, we draw in the concept of “reference object” from classical physics. If the reference objects, the normal samples, are correctly clustered together, we consider the clustering as reasonable; otherwise, we need to tune the parameters in the BRPCA. Tuning the parameters means adjusting the degree of details of component S. The DEFS_w_ algorithm can discover the optimal feature subset size. Considering the unstable stochastic search, we repeated the DEFS_w_ algorithm multiple times with different upper limits of the feature size and selected the feature genes from the DEGs. This may be helpful for selecting out the optimal size of feature genes and getting higher accuracy for discriminating tumor and normal samples. Overall, the proposed approach can identify finer subtypes of colon cancer with fewer feature genes than the other two recent studies and exhibits a generic methodology for identifying cancer subtypes based on gene expression data by common processes.

Inter-tumor diversity of CRC complicates the prediction of disease and treatment outcomes. Subtypes of colorectal cancer identified by classifying gene expression profiles with defined prognostic markers would predict individual patient outcomes more precisely and therefore provide valuable guidance on appropriate therapeutic intervention [45]. It is proposed that CRC subtyping may advance precision diagnostics, treatment, and guide rational drug design. Numerous methods have been attempted to achieve this goal using gene expression datasets [2,4]. In a recent study by Bramsen et al. [46], subtyping strategy was used to CRC transcription profiles for identifying molecular-subtype-specific biomarkers which could contribute to improved patient prognostication. Moreover, other directions have also been taken to find the colorectal subtypes based on pathway profiles, morphological characteristics, clinical and molecular features. Different subtype classifications have been established in recent studies based on three identified molecular pathways: CIN (chromosomal instability), MSI-H (microsatellite instability-high), and CIMP [2,3,47,48,49]. However, there are disagreements among these classifications. There have been many attempts to find consensus in classification of CRC subtypes, and such efforts are essential for revealing prognostic and predictive factors for patient outcomes and to guide treatments [45]. However, no universal subclassification has been agreed upon because of the difficulties and the cost of experimental verification. CRC subtyping consortium (CRCSC) proposed four transcriptional CMSs, which are associated with distinct histopathological features. However, this remains to be further documented, and consensus molecular subtyping is still not in a stage to guide clinical decisions [45]. The reliable molecular subtyping approaches are still needed to unveil clinical potentials.

## Figures and Tables

**Table 1:** Confusion matrix of subtypes identified by our approach and subtypes identified by Muzny et al. [2]. MSI: microsatellite instability; CIMP: CpG island methylator phenotype; CIN: chromosomal instability.

Subtype	S1	S2	S3	S4	S5	S6	S7
MSI/CIMP	1	3	2	2	19	9	22
CIN	24	13	14	2	0	0	2
Invasive	15	1	8	11	1	0	1
Unknown	0	1	2	0	0	0	0

**Table 2:** Confusion matrix of subtypes identified by our approach and subtypes identified by Ren et al. [4].

Subtype	S1	S2	S3	S4	S5	S6	S7
**ECL1**	40	17	26	14	3	0	10
**ECL2**	0	1	0	1	17	9	15

**Table 3:** Overall mean accuracy, overall mean weight accuracy, and mean accuracy for each class by 1000 times of cross-validation using the naive Bayes (NB) algorithm on the feature gene sets.

Cross Validation (%)	Accuracy (%)	Weight Accuracy (%)	Class 1 (%)	Class 2 (%)	Class 3 (%)	Class 4 (%)	Class 5 (%)	Class 6 (%)	Class 7 (%)	Class 8 (%)
10	82.71	84.98	75.70	82.50	91.17	88.50	81.83	90.50	70.67	99.00
20	81.76	82.58	76.72	74.75	78.30	72.25	90.35	83.00	86.17	99.08
30	81.12	81.90	75.90	78.23	76.50	71.40	87.86	82.83	82.50	100.00

## Supplementary Materials

The following are available online at http://www.mdpi.com/2073-4425/9/8/397/s1, Supplementary File 1: The level 3 mRNA gene expression datasets of 153 colon cancers samples and 19 control normal samples. Supplementary File 2: The MATLAB source codes to implement our approach.

## References

[1] Fearon E.R., Vogelstein B. (1990). A genetic model for colorectal tumorigenesis. Cell.

[2] Muzny D.M., Bainbridge M.N., Chang K., Dinh H.H., Drummond J.A., Fowler G., Kovar C.L., Lewis L.R., Morgan M.B., Newsham I.F. (2012). Comprehensive molecular characterization of human colon and rectal cancer. Nature.

[3] Guinney J., Dienstmann R., Wang X., De Reyniès A., Schlicker A., Soneson C., Marisa L., Roepman P., Nyamundanda G., Angelino P. (2015). The Consensus Molecular Subtypes of Colorectal Cancer. Nat. Med..

[4] Ren Z.L., Wang W.H., Li J.M. (2016). Identifying molecular subtypes in human colon cancer using gene expression and DNA methylation microarray data. Int. J. Oncol..

[5] Yiu A.J., Yiu C.Y. (2016). Biomarkers in Colorectal Cancer. Anticancer Res..

[6] Jung S. (2016). In-silico interaction-resolution pathway activity quantification and application to identifying cancer subtypes. BMC Med. Inform. Decis. Mak..

[7] Ma S., Dai Y. (2011). Principal component analysis based methods in bioinformatics studies. Brief. Bioinform..

[8] Ding X., He L., Carin L. (2011). Bayesian robust principal component analysis. IEEE Trans. Image Process..

[9] Liu J.X., Wang Y.T., Zheng C.H., Sha W., Mi J.X., Xu Y. (2013). Robust PCA based method for discovering differentially expressed genes. BMC Bioinform..

[10] Song W.M., Di Matteo T., Aste T. (2012). Hierarchical information clustering by means of topologically embedded graphs. PLoS ONE.

[11] Song W.M., Zhang B. (2015). Multiscale Embedded Gene Co-expression Network Analysis. PLoS Comput. Biol..

[12] Nguyen M.H., Fernando D.L.T. (2010). Optimal feature selection for support vector machines. Pattern Recognit..

[13] Vedaldi A., Zisserman A. (2012). Efficient additive kernels via explicit feature maps. IEEE Trans. Pattern Anal. Mach. Intell..

[14] Luukka P. (2011). Feature selection using fuzzy entropy measures with similarity classifier. Expert. Syst. Appl..

[15] Yu L., Han Y., Berens M.E. (2012). Stable gene selection from microarray data via sample weighting. IEEE/ACM TCBB.

[16] Nguyen X.V., Chan J., Romano S., Bailey J. Effective Global Approaches for Mutual Information Based Feature Selection. Proceedings of the 20th ACM SIGKDD Conference on Knowledge Discovery and Data Mining (KDD’14).

[17] Khushaba R.N., Al-Ani A., Al-Jumaily A. (2011). Feature subset selection using differential evolution and a statistical repair mechanism. Expert Syst. Appl..

[18] Al-Ani A., Alsukker A., Khushaba R.N. (2013). Feature subset selection using differential evolution and a wheel based search strategy. Swarm Evolut. Comput..

[19] Paul S., Das S. (2015). Simultaneous feature selection and weighting—An evolutionary multi-objective optimization approach. Pattern Recognit. Lett..

[20] Draminski M., Rada-Iglesias A., Enroth S., Wadelius C., Koronacki J., Komorowski J. (2008). Monte Carlo feature selection for supervised classification. Bioinformatics.

[21] Kosinski M., Biecek P. (2016). RTCGA: The Cancer Genome Atlas Data Integration. R Package Version 1.2.5. https://rtcga.github.io/RTCGA.

[22] Tumminello M., Aste T., Di Matteo T., Mantegna R.N. (2005). A tool for filtering information in complex systems. Proc. Natl. Acad. Sci. USA.

[23] Matteo T.D., Pozzi F., Aste T. (2010). The use of dynamical networks to detect the hierarchical organization of financial market sectors. Eur. Phys. J. B.

[24] Powers D.M.W. (2011). Evaluation: From Precision, Recall and F-Measure to ROC, Informedness, Markedness & Correlation. J. Mach. Learn. Technol..

[25] Monti S., Tamayo P., Mesirov J., Golub T. (2003). Consensus Clustering: A resampling-based method for class discovery and visualization of gene expression microarray data. Mach. Learn..

[26] Kanehisa M., Goto S., Furumichi M., Tanabe M., Hirakawa M. (2010). KEGG for representation and analysis of molecular networks involving diseases and drugs. Nucleic Acids Res..

[27] Wang X., Zhang L., Li H., Sun W., Zhang H., Lai M. (2016). THBS2 is a Potential Prognostic Biomarker in Colorectal Cancer. Sci. Rep..

[28] Fei W., Chen L., Chen J., Shi Q., Zhang L., Liu S., Li L., Zheng L., Hu X. (2017). RBP4 and THBS2 are serum biomarkers for diagnosis of colorectal cancer. Oncotarget.

[29] Lin X.L., Yang L., Fu S.W., Lin W.F., Gao Y.J., Chen H.Y., Ge Z.Z. (2017). Overexpression of NOX4 predicts poor prognosis and promotes tumor progression in human colorectal cancer. Oncotarget.

[30] Wang R., Dashwood W.M., Nian H., Löhr C.V., Fischer K.A., Tsuchiya N., Nakagama H., Ashktorab H., Dashwood R.H. (2011). NADPH oxidase overexpression in human colon cancers and rat colon tumors induced by 2-amino-1-methyl-6-phenylimidazo[4,5-b]pyridine (PhIP). Int. J. Cancer.

[31] Bauer K.M., Watts T.N., Buechler S., Hummon A.B. (2014). Proteomic and Functional Investigation of the Colon Cancer Relapse-Associated Genes NOX4 and ITGA3. J. Proteome Res..

[32] Sabates-Bellver J., Van der Flier L.G., de Palo M., Cattaneo E., Maake C., Rehrauer H., Laczko E., Kurowski M.A., Bujnicki J.M., Menigatti M. (2007). Transcriptome profile of human colorectal adenomas. Mol. Cancer Res..

[33] Di Pietro M., Sabates Bellver J., Menigatti M., Bannwart F., Schnider A., Russell A., Truninger K., Jiricny J., Marra G. (2005). Defective DNA mismatch repair determines a characteristic transcriptional profile in proximal colon cancers. Gastroenterology.

[34] Sun J., Hu J., Wang G., Yang Z., Zhao C., Zhang X., Wang J. (2018). LncRNA TUG1 promoted KIAA1199 expression via miR-600 to accelerate cell metastasis and epithelial-mesenchymal transition in colorectal cancer. J. Exp. Clin. Cancer Res..

[35] Xu J., Liu Y., Wang X., Huang J., Zhu H., Hu Z., Wang D. (2015). Association between KIAA1199 overexpression and tumor invasion, TNM stage, and poor prognosis in colorectal cancer. Int. J. Clin. Exp. Pathol..

[36] Zhang D., Zhao L., Shen Q., Lv Q., Jin M., Ma H., Nie X., Zheng X., Huang S., Zhou P. (2017). Down-regulation of KIAA1199/CEMIP by miR-216a suppresses tumor invasion and metastasis in colorectal cancer. Int. J. Cancer.

[37] Giaginis C., Georgiadou M., Dimakopoulou K., Tsourouflis G., Gatzidou E., Kouraklis G., Theocharis S. (2009). Clinical significance of MCM-2 and MCM-5 expression in colon cancer: Association with clinicopathological parameters and tumor proliferative capacity. Dig. Dis. Sci..

[38] Hanna-Morris A., Badvie S., Cohen P., McCullough T., Andreyev H.J., Allen-Mersh T.G. (2009). Minichromosome maintenance protein 2 (MCM2) is a stronger discriminator of increased proliferation in mucosa adjacent to colorectal cancer than Ki-67. J. Clin. Pathol..

[39] Byrd J.C., Bresalier R.S. (2004). Mucins and mucin binding proteins in colorectal cancer. Cancer Metastasis Rev..

[40] Nakamori S., Ota D.M., Cleary K.R., Shirotani K., Irimura T. (1994). MUC1 mucin expression as a marker of progression and metastasis of human colorectal carcinoma. Gastroenterology.

[41] Bond C.E., Mckeone D.M., Kalimutho M., Bettington M.L., Pearson S.A., Dumenil T.D., Wockner L.F., Burge M., Leggett B.A., Whitehall V.L. (2016). RNF43 and ZNRF3 are commonly altered in serrated pathway colorectal tumorigenesis. Oncotarget.

[42] Kuhmann C., Li C., Kloor M., Salou M., Weigel C., Schmidt C.R., Ng L.W., Tsui W.W., Leung S.Y., Yuen S.T. (2014). Altered regulation of DNA ligase IV activity by aberrant promoter DNA methylation and gene amplification in colorectal cancer. Hum. Mol. Genet..

[43] Kropotova E.S., Zinovieva O.L., Zyryanova A.F., Dybovaya V.I., Prasolov V.S., Beresten S.F., Oparina N.Y., Mashkova T.D. (2014). Altered Expression of Multiple Genes Involved in Retinoic Acid Biosynthesis in Human Colorectal Cancer. Pathol. Oncol. Res..

[44] Tibshirani R., Hastie T., Narasimhan B., Chu G. (2002). Diagnosis of Multiple Cancer Types by Shrunken Centroids of Gene Expression. Proc. Natl. Acad. Sci. USA.

[45] Bramsen J.B., Rasmussen M.H., Ongen H., Mattesen T.B., Ørntoft M.W., Árnadóttir S.S., Sandoval J., Laguna T., Vang S., Øster B. (2017). Molecular-Subtype-Specific Biomarkers Improve Prediction of Prognosis in Colorectal Cancer. Cell Rep..

[46] Sun W.J. (2016). Molecular subtypes of colorectal cancer: Evaluation of outcomes and treatment. Oncol. Transl. Med..

[47] Hoadley K.A., Yau C., Wolf D.M., Cherniack A.D., Tamborero D., Ng S., Leiserson M.D.M., Niu B., McLellan M.D., Uzunangelov V. (2014). Multiplatform analysis of 12 cancer types reveals molecular classification within and across tissues of origin. Cell.

[48] Roepman P., Schlicker A., Tabernero J., Majewski I., Tian S., Moreno V., Snel M.H., Chresta C.M., Rosenberg R., Nitsche U. (2014). Colorectal cancer intrinsic subtypes predict chemotherapy benefit, deficient mismatch repair and epithelial-to-mesenchymal transition. Int. J. Cancer.

[49] Sadanandam A., Lyssiotis C.A., Homicsko K., Collisson E.A., Gibb W.J., Wullschleger S., Ostos L.C., Lannon W.A., Grotzinger C., Del Rio M. (2013). A colorectal cancer classification system that associates cellular phenotype and responses to therapy. Nat. Med..

